# Effect of retention design of artificial teeth and implant-supported titanium CAD-CAM structures on fracture resistance

**DOI:** 10.4317/jced.52228

**Published:** 2016-04-01

**Authors:** Kristin Ladetzki, Rocío Mateos-Palacios, Agustín Pascual-Moscardó, Eduardo J. Selva-Otaolaurruchi

**Affiliations:** 1 Prosthodontics and Occlusion, University of Valencia, Valencia, Spain; 2 Dental Materials, University of Valencia, Valencia, Spain

## Abstract

**Background:**

For implant-supported hybrid prostheses, high mastication forces and reduced acrylic resin thickness over a metal substructure often cause failures arising from tooth or resin fractures. To assay fracture resistance of artificial teeth and resin in implant-supported hybrid prostheses in relation to the titanium structure and retention design supporting teeth.

**Material and Methods:**

40 specimens bearing incisors were divided into four groups according to the titanium structure supporting the teeth and the type of load force applied: Group I (Control; n=10): Application of static loading to ten incisors set over a metal structure with internal retention. Group II (Control; n=10): Application of static loading to ten incisors set over a metal structure with external retention. The remaining study specimens (n=20) were subjected to 120,000 masticatory and thermal cycles in a chewing simulator. Afterwards, static loading was applied until the point of fracture using an Instron machine. Group III (Study; n=10): Application of dynamic and static loading to ten incisors set over a metal structure with internal retention. Group IV (Study; n=10): Application of dynamic and static loading to ten incisors set over a metal structure with external retention. Data obtained for the four groups was analyzed and compared, determining the type of fracture (cohesive or adhesive) using a reflected light microscope.

**Results:**

Statistical analysis confirmed that there were significant differences in fracture resistance between the four groups. External retention was found to have more fracture resistance than the internal retention.

**Conclusions:**

Hybrid prostheses with titanium substructures and external retention obtained significantly better results than samples with internal retention.

** Key words:**Chewing simulator, thermocycler, fatigue, implant-supported hybrid prosthesis, acrylic teeth, fracture, metal structure design.

## Introduction

Although the number of edentulous patients has declined in recent decades, dentists will always have to treat patients in need of complete rehabilitation resulting from periodontal disease, multiple caries and other causes (1-3). According to Müller *et al.*, the prevalence of edentulism among the elderly in Europe can be as high as 70% depending on the locality, social factors and other determinants (4).

Increasingly, implant-supported prosthetics are being used to treat this patient group as they offer the possibility of rehabilitation by means of a complete fixed prosthesis (5-8). Hybrid prosthetics are generally understood as an implant-supported complete prosthesis screwed onto a minimum of four implants. They consist of a metal substructure covered by resin and acrylic teeth. The patient wears the fixed prosthesis permanently but if necessary it can be removed by the dentist by loosening the screws that fix it to the implants (7,9).

In addition to biological failures, one of the chief problems with complete prostheses is mechanical failure, such as fracture or the debonding of teeth, which occurs more frequently among the incisors because of the functional, non-axial forces to which the front teeth are subjected (10). Patients wearing implant-supported hybrid prostheses present a lower incidence of biological failure but higher rates of mechanical failure caused by elevated masticatory forces and the reduced thickness of the acrylic resin over the metal structure beneath (11).

The bond between the artificial tooth and the resin base is created firstly by the formation of a chemical union between polymethacrylate (PMMA) and the tooth’s radicals and secondly by physical union in the form of mechanical retention between the teeth and the metal substructure, whereby the resin base joins the tooth to the metal. The retention structure can take the form of, for example, indentations drilled in the base of the tooth, a metal post, unpolished, roughened metal surfaces, or slots and holes in the metal structure (12).

The metal structure of the hybrid prosthesis can be manufactured by conventional means –casting in a metal alloy– or by milling using new CAD-CAM technology. Currently, titanium structures fabricated with CAD-CAM techniques are becoming more widely used. The most common designs have internal retention, probably because this is easier to mill. Given the difficulty of milling external retention, an alternative in form of a post is currently being tested. Traditionally, polymethacrylate has been used for fabricating the resin base. According to recent research, chemical union appears to be stronger when thermopolymerizable resins are used rather than self- or photopolymerizable resins (2,10,13).

The debonding of the artificial teeth from the resin base can be classified as either adhesive or cohesive failure. Adhesive failure occurs if there is poor bond strength at the interface between resin and metal. However, cohesive failure indicates that a sturdy union between the resin and metal parts of the prosthetic structure has been established (14).

The metal substructure of the hybrid prosthesis is generally designed with some feature for mechanical retention that helps to stabilize the resin and the teeth. The main aim of this study was to analyze the behavior of artificial teeth set upon titanium substructures in implant-supported hybrid prostheses, assaying fracture resistance in relation to the design of the metal structure (internal or external retention) bearing the teeth.

## Material and Methods

The study included a total of 40 specimens consisting of acrylic sets of upper right incisors (Odilux, Unidesa Odi, Spain) mounted at an angle of 45° over CAD-CAM fabricated titanium structures by means of thermopolymerizable acrylic resin (Idobase High Impact, Unidesa Odi, Spain).

Different metal structure designs were milled using CAD-CAM techniques as follows.

**Figure 1:**
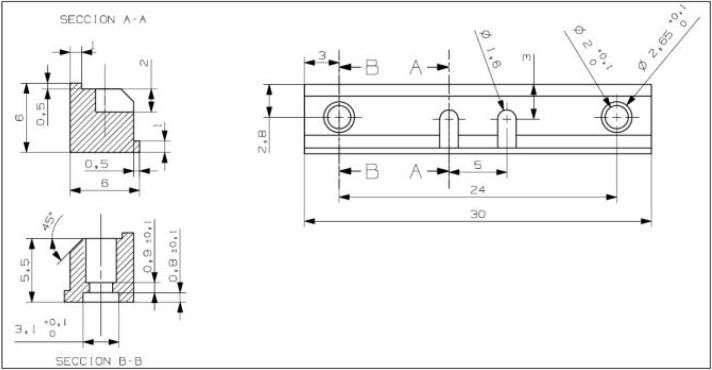
Type A design (internal retention) and Type B design (external retention).

Type A design had internal retention in the form of slots, which are a common feature of structures fabricated using CAD-CAM (Fig. 1). Each bar had two internal retention slots 1.6 mm in width, 2 mm height and 3.6 mm long.

Type B design was made with external retention in the form of posts, which are a common feature of cast hybrid prostheses. Each Type B structure had two external retention pegs with a diameter of 1.6 mm and a height of 2 mm.

The titanium bars were screwed at each end to two BIOMET 3i implants with external connections (OSSEOTITE® NT413, 4 x 13 mm, BIOMET 3i, USA) fixed at an angle of 45° to the test supports by means of epoxy resin (Sadira Epoxi System, Spain).

The correct positioning of the teeth above the titanium bar and the wax-up of the acrylic base was carried out using a plaster index. Sets of acrylic upper right incisors (Odilux, Unidesa Odi; ISO Certificate 22112:2005) were mounted over the titanium structure using thermopolymerizable acrylic resin (Idobase High Impact, Unidesa Odi). The incisors mounted on bars with positive retention were prepared by drilling holes into the base of each tooth. Firstly, the position of each hole was marked with ink, placing the tooth and the titanium bar in the plaster index, and secondly, drilling the hole with a 2.3 mm diameter tungsten drill bit in the correct position.

The 40 samples were divided into four groups.

Group I (Control): 10 Type A design samples (internal retention) were subjected to static loading to the point of fracture using an Instron machine (INSTRON 4411, USA).

Group II (Control): 10 Type B design samples (external retention) were subjected to static loading to the point of fracture using an Instron machine (INSTRON 4411, USA).

In addition, study groups were tested using a chewing simulator with dynamic loading in combination with a thermocycler (CS-4.2, THE-1100, SD-Mechatronik, Germany); specimens were subjected to fatigue testing under controlled humidity conditions, changing the water temperature by intervals.

Group III (Study): 10 Type A design samples (internal retention) were subjected to 120,000 masticatory dynamic load cycles in the chewing simulator / thermocycler and then subjected to static loading until the point of fracture using the Instron.

Group IV (Study): 10 Type B design samples (external retention) were subjected to 120,000 masticatory dynamic load cycles in the chewing simulator / thermocycler and then subjected to static loading until the point of fracture using the Instron.

Groups III and IV were subjected to 120.000 masticatory cycles under a weight of 5 kg, which corresponds to approximately six months of natural mastication. A steel point with 2 mm diameter was used as the antagonist. This was positioned in contact with the palatine face of the incisor to simulate the non-axial forces to which natural upper incisors are subjected.

**Figure 2:**
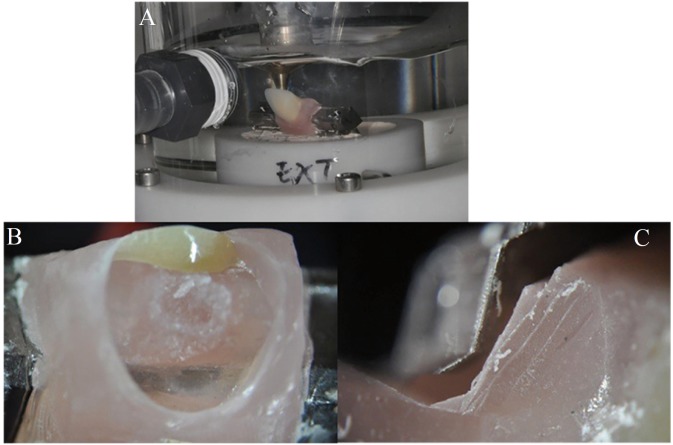
A) Chewing simulator-thermocycler. B) Cohesive failure. C) Adhesive failure.

During fatigue testing, the thermocycler created a wet environment in order to subject the samples to thermal stress, to age the teeth and the resin. The temperature of the water in contact with the samples was raised from 5° to 55° in intervals of 60 seconds with pauses of 12 seconds between intervals (Fig. 2A). A total of 622 thermocycles were applied.

After fatigue testing, the state of the samples was checked, looking for cracks and/or cohesive or adhesive failure, using a reflection microscope (Nikon SMZ-10A, Japan).

When dynamic load testing had been completed, samples were subjected to static load testing in the Instron machine, with the steel antagonist set at a velocity of 1mm/min maintained until the point of fracture. Afterwards, the type of fracture (adhesive or cohesive) was determined under the microscope.

-Statistical Analysis

Statistical analysis was performed applying the Kruskal-Wallis test to determine the homogeneity of force distribution across the four sample groups. Then the Mann-Whitney test was used to identify differences between groups. The significance level was established as 5% (*p*=<0.05).

## Results

**Figure 3:**
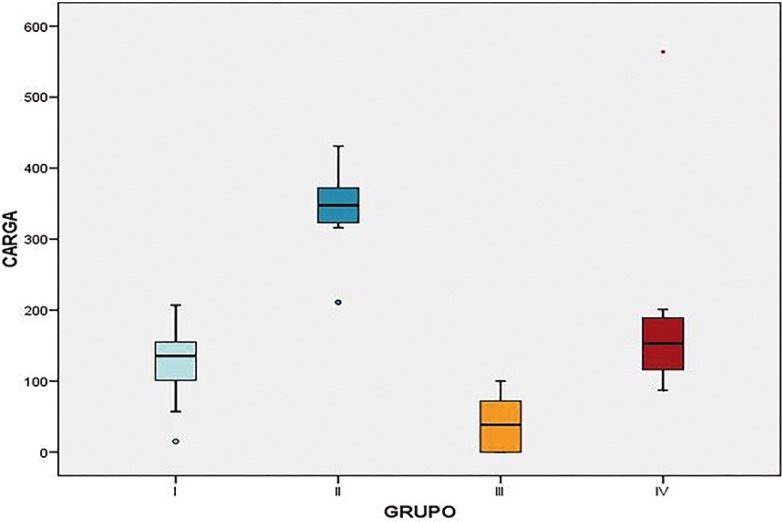
Distribution of breakage forces (in Newtons) after applying static loading to all four groups using the Instron machine.

Statistical analysis confirmed that there were significant differences in fracture resistance between the four groups ( Table 1). As shown in figure 3, the Type B design (external retention) was found to have more fracture resistance than the Type A design (internal retention). It was also found that the study groups that had been tested by means of the chewing simulator and thermocycler showed lower resistance to fracture than their respective control groups.

**Table 1:**
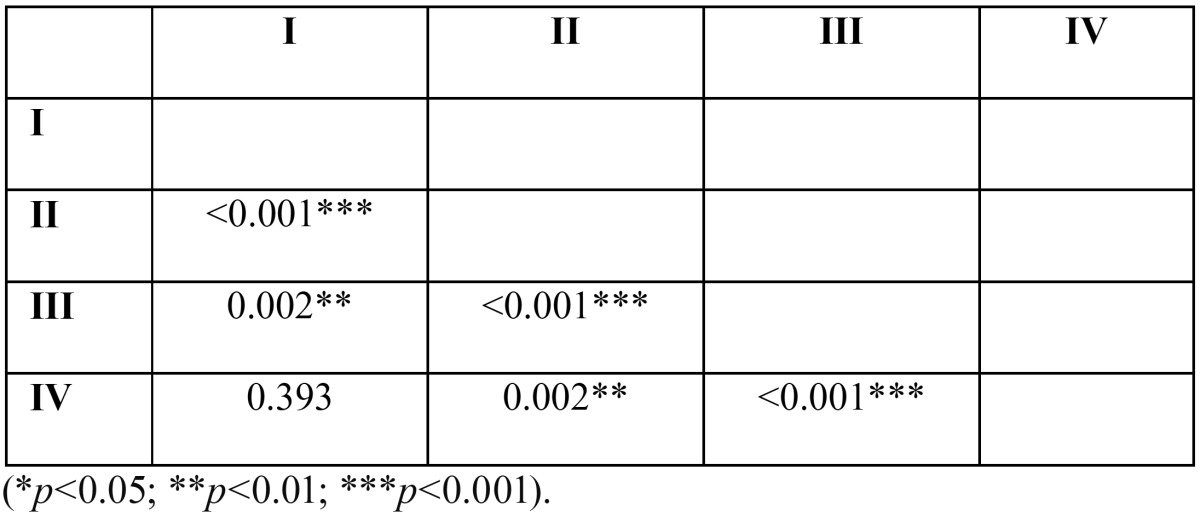
Statistically significant differences between the four groups.

After subjecting samples to static loading, cohesive fractures were detected in all Type B design specimens, which showed that there was a close union between the prosthesis’s artificial teeth, resin base and metal structure (external retention) (Fig. 2B). However, all Type A design specimens (internal retention) presented adhesive fractures, showing less strength in the union between the resin base and the metal structure (Fig. 2C).

## Discussion

Like all types of treatment, hybrid prosthetics have both advantages and disadvantages. One of the most important advantages is that hybrid prostheses, supported by a small number of implants, provide the opportunity for edentulous patients to enjoy fixed prostheses at a reasonable cost. If the prosthesis needs repair at a later stage it can be easily removed by the dentist. One of the disadvantages is the high mechanical failure rate such as fracture or debonding of artificial teeth which result from the forces generated by mastication.

Nevertheless, in spite of the disadvantages, hybrid prosthetics offer a valid alternative to mucosupported removable rehabilitations or the more costly implant-supported ceramic structures (7,9,15).

There are currently many types of artificial prosthetic teeth available on the market, including conventional acrylic teeth, acrylic teeth with interpenetrating polymer networks (IPN) and composite resin teeth (16,17). Combining conventional acrylic resins with IPNs will create a structure with a three-dimensional network of two intertwining polymers. This offers more resistance to wear and tear, a more stable color and greater masticatory efficiency compared to conventional acrylic teeth, but without compromising its capacity to form a strong bond with the resin base (18). This material continues to be the most widely used for fabricating complete prosthesis.

Composite resin with inorganic silica fillers is another material used for fabricating artificial teeth. Because of the inorganic filler, these teeth have better mechanical properties, including greater hardness and higher abrasion resistance, but according to some research they show lower bond strength to the resin base. Another disadvantage would appear to be an increased elastic modulus which causes greater transmission of masticatory forces to the supporting prosthetic structure, producing both adhesive and cohesive fractures (16,19). For these reasons, it was decided to opt for IPN teeth in the present study.

Traditionally, polymethacrylates have been used to fabricate the resin base in hybrid prostheses. According to recent studies, the chemical bond would appear to be higher when thermopolymerizable resins are used than self- or photopolymerizable resins (2,20).

Since the launch of the Willytec® chewing simulator in 1997, this has become the most widely used testing device all over the world. The chewing simulator/thermocycler used in the present study (CS-4.2, THE-1100, SD-Mechatronik, Germany) also employs Willitec® technology. In order to simulate an average term in the mouth, and in accordance with most of the literature, the present study applied 120,000 masticatory cycles, the equivalent of six months functional life in the mouth (21-23). Apart from the vertical movement involved in mastication it also makes a 0.7mm horizontal movement, simulating the sliding of the mandible from side to side during mastication.

It was decided to set the study specimens at an angle of 45° in order to create an interincisal angle of 135°, which is the angle found in natural dentition. During fatigue testing, the thermocycler created wet conditions, changing the temperature of the water in contact with the samples between 5° and 55° to imitate the temperature changes that can take place in the mouth as we eat and drink, which have been documented in numerous studies (24).

Fatigue testing was performed applying a force of 49N. The literature contains varying data on the masticatory forces to which the incisors are subjected; averages range between 25N y 50N, although parafunctional mastication can generate higher forces (25).

Various studies have shown that it is easier to create prosthetics that achieve adequate passive fit using CAD-CAM techniques then using casting techniques (26-28). It is also an established fact that the acrylic part of the hybrid prosthesis requires a minimum thickness of 1.5-2 mm in order to avoid increasing the risk of fracture. It is generally stated in the literature that retention devices are needed to form a strong bond with the metal part of the prosthetic structure (26). However, as far as the authors of this study are aware, apart from a few clinical case studies, there is no published research dealing with the subject of the present study –retention design– and so there are no statistical data with which to compare the present findings.

The present study observed statistically significant differences between the prosthetic designs. A large number of the samples of type A design with internal retention failed to withstand average range masticatory forces, while type B samples withstood higher forces.

In addition to differences between the designs in relation to fracture resistance, it was also found that specimens subjected to wear and tear and thermal stress by means of the chewing simulator/thermocycler fractured under significantly less force compared to the control groups.

## Conclusions

It may be concluded that the design of the metal substructure influences the union between teeth and the base of the hybrid prosthesis.

Hybrid prostheses with metal substructures designed with external retention show significantly better fracture resistance than designs with internal retention.

Fracture resistance of the artificial teeth and the resin base decreased significantly in fatigue testing in the chewing simulator/thermocycler, samples with internal retention having less resistance to forces equivalent to average range functional loads in the mouth.

These findings confirm impressions received in clinical practice that patients wearing hybrid prostheses suffer a high rate of mechanical failure with the most commonly used designs with internal retention.

It is hoped that in the future it will be possible to choose between a wider variety of CAD-CAM designs and that this topic becomes a subject of further research in the aim of reducing the number of mechanical failures in hybrid prostheses.
